# Renal Casts in Phthisis

**Published:** 1910-03-26

**Authors:** 


### Medicine.

### RENAL CASTS IN PHTHISIS.

In the earlier days of urinary examinations, when there were no elaborate centrifugal machines which make the detection of even a single cast in a bulk of urine comparatively easy, the discovery of renal tube casts in a urinary deposit microscopically could be regarded as proof positive of important nephritis. The refinements of clinical methods have now become so great, however, that casts are often discovered in cases in which it would be misleading to lay any stress upon them at all. In a sense it is a pity that one has been deprived of so simple a test as that which might be summarised by saying: "If there are casts under the microscope the case is one of nephritis"; but as the test from being so simple has become complex, it behoves us to study the matter and see what stress can still be laid upon the finding of casts in the urine.

Some extensive researches upon the subject have been published by Dr. Montgomery in the Fourth Annual Report of the Phipps Institute. The condition of the urine as regards albumin and renal tube casts was systematically investigated in a large consecutive number of phthisical patients. It is quite true, as Walsh has shown, that very few patients dying from phthisis fail to exhibit in histological preparations of their kidneys either actual tubercles or foci of inflammation, degeneration, or necrosis of the epithelium. So universal are these changes, however, that they cannot be given the clinical value that is ordinarily carried by the term nephritis. Nevertheless Walsh's researches prepare one for the fact that albumin and casts are both of common occurrence in phthisical patients.

Strictly speaking, no doubt there is a modicum of nephritis in all such cases. The question is, Out of so many how is one to pick those in which the nephritis matters from those in which it does not? In other words, how is one to determine when either albumin or renal tube casts, or both, are to be interpreted as serious, and when they may be regarded as of little significance in any particular case?

Montgomery's figures are based upon over five hundred cases, and they seem to indicate that small quantities of albumin occur in the urines of between one-third and one-half of all decidedly phthisical patients; that large quantities are uncommon; that albumin may occur without renal tube casts, in which case it is not very important; that it may occur with tube casts, and further that renal tube casts may occur without albuminuria. This latter possibility is one that is not generally recognised, and we shall refer to it again later. According to Montgomery the constant occurrence of fairly large numbers of casts in tuberculosis, even when albumin is absent or present in small amounts, is of bad prognostic omen. Casts are constantly present in most cases for a few weeks before death. The constant absence of casts, on the other hand, is to a certain extent a sign of favourable prognosis.

The original paper is well worth reading. The following are Montgomery's own conclusions:—

"Albumin is present in about one-third of the cases of pulmonary tuberculosis coming to the Phipps Institute dispensary and hospital. These consist mostly of moderately and far advanced cases. In about one-half of these cases of albuminuria the albumin occurs in association with casts; albumin is present in about one-half of the hospital cases at the time of admission. It is present in nearly all specimens in the last few weeks of life, and in all cases during the last few weeks of life that we have especially investigated.

"The amount of albumin in the majority of patients with albuminuria, even of those patients in the last few weeks of life, is only a trace. Only exceptionally do patients have enough albumin to be estimated with the Esbach albuminometer, but as much as 2.6 per cent. has been observed. Marked variations in the amount of albumin may occur during the twenty-four hours. In one case specially studied the largest amount of albumin found occurred between 12 noon and 6 p.m., and the smallest amounts between 12 midnight and 6 a.m.

"Albumin in large amounts is rare in tuberculosis, unless in association with casts or with pus or blood. Albumin in large amounts occurred more frequently in cases in which the autopsy revealed many intestinal ulcers than in those with few or no ulcers.

The occurrence of albumin and casts in the urine of tuberculous patients at the Phipps Institute is apparently independent of any dietetic treatment.

" Urinary casts, like albumin, are present in about one-third of the cases of pulmonary tuberculosis at the Phipps Institute at the time of admission. In a little over half of these cases with casts albumin is also present; in the remainder it is absent. Casts are present in about three-fourths of the hospital cases at the time of admission. Albumin occurred in only about one-half of these cases. In all cases during the last few weeks of life, and in nearly all specimens, casts, like albumin, were present.

" In cases with casts in the urine one cast may be present or many. Very large numbers are seen chiefly in cases of marked nephritis, and in patients during the last few days of life. Many casts at times occur in the absence of albumin.

### Varieties of Casts.

" Any variety of cast may be found in tuberculosis. The commonest varieties in our experience are the granular and hyaline. In advanced cases we have observed the granular cast more often than the hyaline. Casts showing cells are nearly as frequent in advanced cases as are the hyaline. These casts for the most part show only a few cellular attachments. In some cases in the last few days of life large numbers of hyaline casts make their appearance, while granular casts and free cellular elements are very few in number or entirely absent. These large numbers of hyaline casts are present at times in association with only a trace of albumin, at others with a considerable amount.

" Leucocytic casts, in the sense of casts composed entirely of leucocytes, are rare in my experience. When they appear as attachments to casts it is often difficult to differentiate them from epithelial cells. Red blood casts have been observed only a few times. Amyloid casts were observed in the urine of four cases in the course of the year. Two of these cases ended fatally, and in each the liver at autopsy revealed amyloid change. The specific gravity of the four specimens of urine was never above 1012, the albumin was always considerable in amount, and casts were always numerous. Sugar and diazo-reactions were absent.

### Their Prognostic Value.

" We believe that the constant presence of fairly large numbers of casts in the urine in pulmonary tuberculosis is of unfavourable prognostic import, and that the constant absence of casts is of rather favourable prognostic import. The occurrence of both albumin and casts in the urine is of more significance, as a rule, than either of these findings alone. Both albumin and casts, however, at times occur to quite a marked degree in patients who show no other symptoms than those exhibited by many patients who have little or no albumin, and few or no casts. Albumin and casts in the urine usually become more marked as the disease progresses, and often diminish or disappear when the patient's condition improves. Albumin and casts in pulmonary tuberculosis are not often associated with other evidences of nephritis.

" Albumin and casts in tuberculosis are more frequent in association with certain symptoms and conditions than with others, though it is often impossible to tell how much they depend on these conditions. They occur considerably more frequently than usual in cases of diarrhœa, intestinal involvement diagnosed clinically, and arterio-sclerosis; also in cases with a urinary specific gravity over 1018, with tubercle bacilli in the sputum, and in progressive cases or cases showing extensive pulmonary involvement. In cases with anorexia, gastric disturbance, accentuated second sound at the aortic cartilage, and œdema, albumin and casts occur a little more frequently than in cases not presenting these symptoms. Albumin occurs most frequently in the period under ten years, and casts in the period over fifty years. Casts alone and casts in association with albumin occur considerably more often in the male than in the female sex. Albumin, according to the combined statistics of the last and the current year, appears in only a very slightly larger proportion of males than females."

